# Special Issue: Advance in Energy Harvesters/Nanogenerators and Self-Powered Sensors

**DOI:** 10.3390/nano12183167

**Published:** 2022-09-13

**Authors:** Qiongfeng Shi, Jianxiong Zhu

**Affiliations:** 1 Joint International Research Laboratory of Information Display and Visualization, School of Electronic Science and Engineering, Southeast University, Nanjing 210096, China; 2 School of Mechanical Engineering, Southeast University, Nanjing 211189, China

Internet of things (IoT) technologies are greatly promoted by the rapidly developed 5G-and-beyond networks, which have spawned diversified applications in the new era including smart homes, digital health, sports training, robotics, human–machine interaction, metaverse, smart manufacturing and industry 4.0, etc. To accommodate the enormous and widely distributed power demands needed for IoT nodes, self-sustained systems enabled by energy harvesting technologies (e.g., piezoelectric, triboelectric, electromagnetic, pyroelectric, thermoelectric, photovoltaic, etc.) have emerged as a promising solution. In recent decades, we have witnessed the rapid advancement of energy harvesters and nanogenerators in terms of innovations in materials, mechanisms, structural designs, etc., leading to ever-increasing energy conversion efficiency and output power performance. Except for directly serving as energy harvesters and power sources, energy harvesting devices can also be used as self-powered sensors to effectively lower the power consumption of a system, due to their self-generated signals corresponding to the external stimuli. Therefore, the advancement of energy harvesters and self-powered sensors could push forward the realization of self-sustained systems with continuous functional operations.

This Special Issue includes 10 research and review articles in the field that showcase the recent advances in energy harvesters, nanogenerators, and self-powered sensors for various applications, which should give readers a glimpse of the challenges, opportunities, and development trends of energy harvesting technologies.

There are six research articles from researchers across the world that explore device innovations in energy harvesting and self-powered sensing applications. To boost the conversion efficiency, Zhai et al. reported on an energy harvester using a triboelectric–electrostatic coupling mechanism that showed a 46-fold enhancement compared to a single triboelectric mechanism [1]. With this high output performance, a self-powered system was successfully demonstrated using gait recognition for access control. To effectively scavenge energy from low-frequency human motions, Tang et al. presented a non-resonant energy harvester that hybridized piezoelectric–electromagnetic–triboelectric in a synergic design [2]. In practical applications, the energy harvester was deployed on various body parts to show its energy generating capability for powering a wireless IoT sensor node. Then, Huang et al. investigated the self-powered resistance-switching ability of a triboelectric nanogenerator on resistive random access memory (RRAM) [3]. The RRAM exhibited excellent resistance-switching performance when driven by the triboelectric nanogenerator, with a switching ratio up to 2 × 10^5^.

In terms of self-powered sensing, Labed et al. developed a self-powered photodetector using an InZnSnO/β-Ga_2_O_3_ Schottky barrier diode [4]. Operated in the self-powered mode at 0 V, the device achieved a high photo-to-dark current ratio (3.70 × 10^5^) and a good photoresponsivity (0.64 mA/W). Based on a Ag_2_O/β-Ga_2_O_3_ heterojunction, Park et al. proposed a self-powered deep ultraviolet photodetector [5]. When operated at 0 bias voltage, a high photoresponsivity of 12.87 mA/W was obtained, showing good potential for use in ultraviolet sensing systems. Next, Lin et al. constructed a new light-driven integrated bio-capacitor based on bacteriorhodopsin and artificial nanochannels [6]. Microfluidic chips and a single nanopore structure were integrated to make the bio-capacitor more stable, and the photocurrent duration time was effectively regulated by varying the single nanopore’s size.

Meanwhile, for those readers that are seeking an overview of the progress of energy harvesting technologies, four review articles covering different aspects of the field are also included in this Special Issue. Delgado-Alvarado et al. reported on the recent progress of nanogenerator technologies, including piezoelectric, electromagnetic, thermoelectric, and triboelectric, in terms of materials, applications, challenges, and future prospects for green energy harvesting [7]. Then, Wang et al. systematically reviewed the progress of implantable biomedical devices (i.e., biosensors, energy harvesters, and stimulation therapy devices) based on triboelectric nanogenerators, and discussed the remaining challenges and opportunities for the aspects of multifunctional materials and self-sustained close-looped systems [8]. Haroun et al. summarized the recent progresses in using triboelectric nanogenerators and hybridized generators for vibration energy harvesting and monitoring, with detailed analyses of the working mechanism, design principle, output performance, and important applications [9]. To manage the increasingly serious arsenic pollution in the living environment and maintain a healthy and beautiful ecosystem for human beings, Hu et al. reviewed the advances in electrode systems based on nanomaterials and their performance in arsenic detection [10]. By using silicon and its compounds, as well as novel polymers, for the detection of arsenic detection, such as noble metals, bimetals, other metals, and their compounds, such as carbon-, nano-, and biomolecules, they showed new routes for investigating novel nanomaterial sensing.

We would like to thank all the authors and reviewers for their contributions to this Special Issue. We also hope that the articles showcased here are interesting and helpful for readers and can inspire new innovations in the field.
